# Flow-enhanced solution printing of all-polymer solar cells

**DOI:** 10.1038/ncomms8955

**Published:** 2015-08-12

**Authors:** Ying Diao, Yan Zhou, Tadanori Kurosawa, Leo Shaw, Cheng Wang, Steve Park, Yikun Guo, Julia A. Reinspach, Kevin Gu, Xiaodan Gu, Benjamin C. K. Tee, Changhyun Pang, Hongping Yan, Dahui Zhao, Michael F. Toney, Stefan C. B. Mannsfeld, Zhenan Bao

**Affiliations:** 1 Department of Chemical Engineering, Stanford University, Stanford, California 94305, USA; 2 Stanford Institute for Materials and Energy Sciences, SLAC National Accelerator Laboratory, 2575 Sand Hill Road, Menlo Park, California 94025, USA; 3 Advanced Light Source, Lawrence Berkeley National Laboratory, Stanford, Berkeley 94720, USA; 4 College of Chemistry, Peking University, Beijing 100871, China; 5 Stanford Synchrotron Radiation Lightsource, SLAC National Accelerator Laboratory, Menlo Park, California 94025, USA; 6 Center for Advancing Electronics Dresden, Dresden University of Technology, 01062 Dresden, Germany; 7 School of Chemical Engineering, Sungkyunkwan University (SKKU), Suwon 440-746, Republic of Korea

## Abstract

Morphology control of solution coated solar cell materials presents a key challenge limiting their device performance and commercial viability. Here we present a new concept for controlling phase separation during solution printing using an all-polymer bulk heterojunction solar cell as a model system. The key aspect of our method lies in the design of fluid flow using a microstructured printing blade, on the basis of the hypothesis of flow-induced polymer crystallization. Our flow design resulted in a ∼90% increase in the donor thin film crystallinity and reduced microphase separated donor and acceptor domain sizes. The improved morphology enhanced all metrics of solar cell device performance across various printing conditions, specifically leading to higher short-circuit current, fill factor, open circuit voltage and significantly reduced device-to-device variation. We expect our design concept to have broad applications beyond all-polymer solar cells because of its simplicity and versatility.

## Abstract (web summary)

Solution printing is a desirable route for manufacturing organic solar cells, whilst the major challenge lies with morphology control. Here, Diao *et al.* use a microstructured blade to guide the solution flow during printing, which improves polymer crystallization and the resulting device performance.

## Graphical abstract

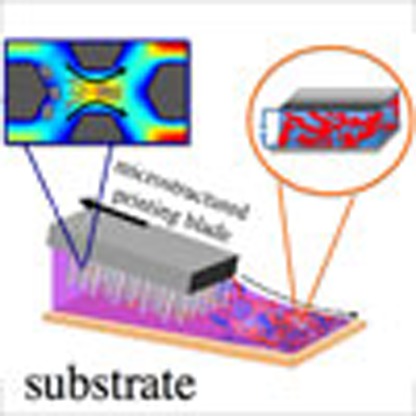

Solution printing is an attractive alternative for realizing energy-efficient, high-throughput, low-cost and low carbon-footprint manufacturing of photovoltaics. This approach has the potential to meet the rapidly growing demand for energy, known as the ‘one-gigawatt-per-day' challenge^1^. However, several outstanding challenges need to be addressed to realize the full potential of printing. A key problem lies in the lack of control of solar cell morphology during solution printing, an issue encountered not only during the printing of organic solar cells^2^,^3^,^4^, but also perovskite^5^,^6^,^7^ and thin film solar cells^8^. It is well known that the efficiency of organic solar cells critically depends on the thin film morphology^9^,^10^,^11^,^12^. For organic bulk heterojunction (BHJ) solar cells, previous studies have shown that the domain size of the phase-separated structures^13^,^14^,^15^,^16^, degree of crystallinity^10^,^17^,^18^,^19^, interfacial orientation^20^ and the presence of mixed phases^16^,^21^ are among the important morphological characteristics collectively affecting exciton transport and dissociation, as well as charge transport, recombination, collection and ultimately power conversion efficiency (PCE).

Recently, there is increasing evidence suggesting that, for some BHJs, higher crystallinity of the polymer donor phase can lead to an improved PCE by increasing both the short-circuit current and the fill factor^10^,^17^,^18^,^19^,^22^. Increased crystallinity may also lead to a longer exciton diffusion length^23^,^24^,^25^ and a higher absorption coefficient^26^, both of which can increase the short-circuit current. Higher crystallinity also improves the charge carrier mobility, thereby facilitating charge collection and enhancing the fill factor^22^. To improve polymer crystallinity, commonly used methods include post-deposition thermal or solvent vapour annealing^12^ and the employment of high-boiling point cosolvent additives^27^. Alternatively, nucleation agents have also been used to accelerate crystallization rate by inducing heterogeneous nucleation^28^. However, increasing the polymer crystallinity can negatively impact on the PCE when the increase in crystallinity comes at the expense of increasing the domain size at the same time^29^. Increasing the domain size much beyond the exciton diffusion length (typically 10–20 nm) is undesirable due to the resulting higher exciton recombination rate^15^,^30^. In particular, all-polymer solar cells have been under rapid development recently due to their advantages over polymer-fullerene solar cells in the versatility of molecular design and the enhanced chemical and morphological stabilities^15^,^31^,^32^,^33^,^34^,^35^. However, non-ideal thin film morphology represents a major factor limiting attainable device efficiencies, especially the low crystallinity and large domain sizes observed in recent reports^15^,^30^,^35^.

In this work, we introduce a novel approach for directing microphase separation, in particular polymer crystallization, by manipulating the fluid flow during solution printing of BHJ solar cells using microstructured printing blades (hereafter referred to as FLUENCE, or fluid-enhanced crystal engineering). The aim of this method is to enhance the polymer crystallinity without increasing the domain size by a mechanism involving flow-induced nucleation. This method takes advantage of the unique flow characteristics of meniscus-guided coating techniques, such as solution shearing^36^,^37^,^38^,^39^,^40^ and roll-to-roll printing^2^,^3^,^4^, and is therefore distinct from previously reported morphology control methods^11^,^12^,^41^. We demonstrate that our flow-enhanced solution printing method is able to substantially increase the degree of crystallinity of the printed all-polymer solar cells, while at the same time reducing the domain size of the phase-separated structure to bring it closer to the length scale of the expected exciton diffusion length, leading to improved PCE.

## Results

### Flow design for enhancing polymer crystallization

**Figure 1:**
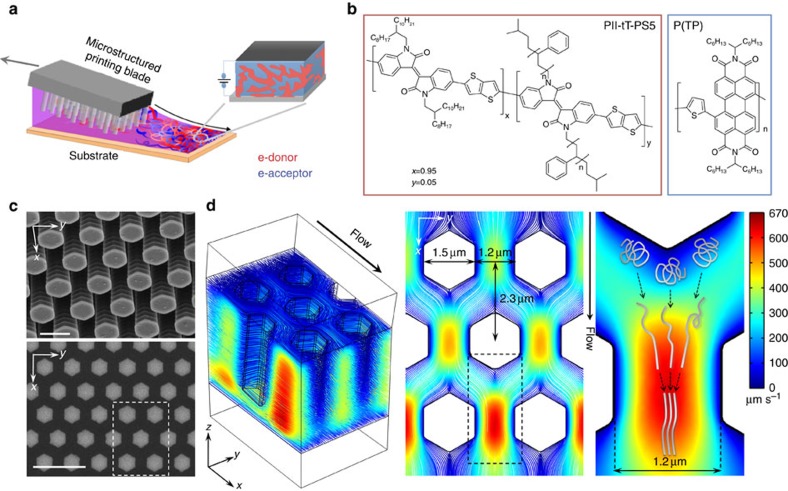
FLUENCE for controlling microphase separation of printed all-polymer solar cells. FLUENCE stands for ‘fluid-enhanced crystal engineering'. (**a**) Schematic of the FLUENCE method implemented on the solution shearing platform. (**b**) Schematic of the microphase-separated morphology in bulk heterojunction solar cell and the molecular structures of the electron-donor and electron-acceptor polymers used in this study. (**c**) Scanning electron microscope images of the microstructured printing blade, scale bar 2 μm (top), 5 μm (bottom). The white dotted line indicates the size of the simulation box in the *xy* plane. (**d**) Finite element simulation results (stream-line representation) of the flow field between the microstructured printing blade and the substrate. The simulated printing speed is 50 μm s^−1^. The colour scale of the fluid velocity is shown to the right. In this case, the flow is mainly driven by solvent evaporation instead of the printing motion. The cut plane shown (middle image) lies parallel to the substrate, approximately equidistant to the blade and the substrate in the *z* direction. The hypothesized polymer conformation change, alignment and aggregation/crystallization under extensional and shear flow are depicted in the simulated flow field (right image).

Herein we describe the design concept of FLUENCE for controlling polymer crystallization and therefore microphase separation in BHJ solar cell systems. We previously demonstrated the use of FLUENCE for large-area coating of aligned single-crystalline arrays of small molecule organic transistors^38^. In this work, however, the flow design is based on an entirely different concept (discussed below) given that polymer crystallization is strongly influenced by chain conformation dynamics, distinct from small molecules. The effect of fluid flow on polymer phase behaviour has been studied extensively in the field of polymer rheology, in particular for bulk commodity polymers such as polypropylene^42^,^43^,^44^ and recently, biomolecules such as DNA^45^. However, these concepts have not been explored for the solution printing of solar cell materials. Flow-induced nucleation has been observed in dilute polymer solutions at concentrations (∼2 wt%) and shear rates (4–40 s^−1^) comparable with those of our processing conditions^46^,^47^. This phenomenon is closely related to flow-induced changes in polymer conformations. In particular, flow-induced chain extension and alignment are deemed responsible for expedited polymer crystallization due to a lowered entropic barrier to the formation of ordered structures^44^. Among the various flow types, extensional flow has been shown to be the most effective in inducing crystallization by means of stretching the polymer chains^42^,^43^,^44^; shear flow was also found to promote crystallization kinetics, although much less effectively, by possibly increasing chain alignment^44^,^48^,^49^. In meniscus-guided solution coating methods^40^, such as the solution shearing method^36^,^37^,^38^,^50^ used in this study, shear flow is the dominant flow type with minimal extensional flow characteristics. To induce extensional flow as well as to increase the shear rate across various printing conditions, we pattern the printing blade with micropillar arrays, which ‘comb' the ink during the printing process to direct the microphase separation between the polymeric electron donor and acceptor materials (Fig. 1a). Finite element-based fluid simulation results show that the presence of micropillars effectively induced extensional flow and enhanced the shear rate (Fig. 1d). Using the pillar arrays shown in Fig. 1c, the maximum extensional strain rate (δ*v*/δ*x*) increased by ∼2 orders of magnitude to ∼500 s^−1^, and the maximum shear rate (δ*v*/δ*y*) increased by ∼40 times to over 1,000 s^−1^ as compared with the case of the unstructured blade. These enhancements are attributed to several key design parameters deduced from fluid simulations. First, small pillar spacing along *y* axis (perpendicular to the shearing direction) is critical to expediting the flow in between the pillars and for inducing high shear rates. Second, the staggered arrangement of the pillar array as well as the close row spacing along *x* axis are important for generating a high extensional strain rate in the direction of the flow (Fig. 1d). We hypothesize that the high extensional strain rate facilitates stretching of the polymer chains, which are subsequently aligned under high shear rate (Fig. 1d). Both effects cooperate to promote polymer nucleation and drive microphase separation^10^ between the donor and acceptor phase (Fig. 1b). To verify our design concept, we later show that increasing the pillar gap and the row spacing by over tenfold diminishes the effect of FLUENCE on film morphology (see Discussion). It is worth noting that evidence of flow-induced crystallization have been presented and studied in depth in the context of isotatic polypropylene crystallization from melt^44^,^51^,^52^,^53^. Lamberti *et al.* have shown that flow-induced crystallization is due not only to extensional flow, but also to shear flow-induced orientation ordering, which has been observed in melt. The flow-induced orientation decreased the entropy of phase change shown using computational approach. Their studies further support our design concept and hypothesis.

To implement FLUENCE, a micropillar-patterned printing blade was fabricated using photolithography and reactive ion etching (Methods). The patterned blade was subsequently functionalized with an n-octadecyltrichlorosilane monolayer to minimize polymer deposition on the blade during printing. Using FLUENCE, the active layers were printed at various printing speeds from their chlorobenzene solutions on a zinc oxide electron transport layer spin-coated onto indium-doped tin oxide (ITO)/glass. Using unstructured blades, reference films were made at the same conditions to compare with the FLUENCE-printed films (see Methods for details). The polymer donor we use is the poly(isoindigo-thienothiophene)-based conjugated polymer with 5 mol% low molecular weight polystyrene (PS) side chains (*M*_n_=1,300 g mol^−1^) via random copolymerization (referred to as PII-tT-PS5)^54^. The acceptor system is a perylene tetracarboxlic di-imide containing polymer (referred to as P(TP)) (Fig. 1b). The molecular design concept was described in our previous work^15^,^34^.

### Degree of crystallinity analysis via GIXD

**Figure 2:**
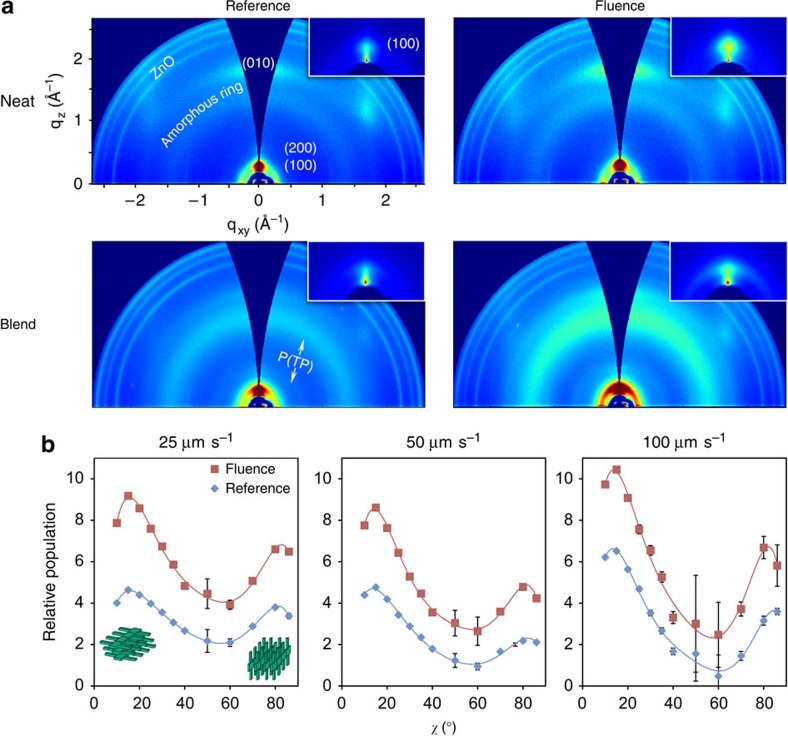
Polymer crystallinity analysis via GIXD. (**a**) Comparison of the diffraction patterns between the FLUENCE-printed and the reference films for neat donor polymer films and the blend films. The π–π stacking peak and the lamella peaks are labelled as (010) and (100) to (200), respectively. (Inset) Magnified images of the (100) peak (geometrical correction not applied here so as to clearly show the intensity difference). Across all images, the intensity is scaled by exposure time and the irradiated volume, to allow visual comparison of the peak intensities. Films were printed at 25 μm s^−1^ from 7 mg ml^−1^ chlorobenzene solution at 50 °C. The average film thickness was 124 nm. GIXD was taken with the printing direction of the films oriented parallel to the incident beam (shown here) as well as perpendicular to the incident beam (Supplementary Fig. 3). (**b**) Comparison of geometrically corrected orientation distribution functions at various printing speeds (25–100 μm s^−1^) in neat donor polymer films. The geometrical correction was performed on pole figures shown in Supplementary Fig. 2. The corrected intensity of the (010) peak, or sin(*χ*)*I*(*χ*), represents the relative population of the crystallites with a particular orientation *χ*, the polar angle (Supplementary Fig. 1). In this plot, *χ*=0° indicates face-on orientation and *χ*=90° indicates edge-on orientation. The relative orientation of the crystallite at corresponding *χ* is shown as inset. The red and blue curves correspond to films printed with and without FLUENCE, respectively. The relative degree of crystallinity is obtained by integrating the area below each curve. From left to right, the rdoc is 89%, 94%, 87%, respectively. The error bars were from the s.d.s from the fitted (010) peak areas (Supplementary Fig. 1). For most data points, the error bars are too small to be visible.

First, we characterize how FLUENCE alters polymer crystallinity in printed thin films using grazing incidence X-ray diffraction (GIXD). The crystallinity of the blend films is relevant for the donor polymer PII-tT-PS5, since the acceptor polymer P(TP) remains amorphous at all tested conditions^15^ and only contributes to the amorphous halo in the GIXD patterns (Fig. 2). Most strikingly, both the π–π stacking peak (010) and the lamella stacking peak (100) of the donor polymer exhibit substantially higher intensities in FLUENCE-printed films, for both neat donor polymer films and the blend films. This qualitative observation indicates that our flow design has effectively enhanced the degree of crystallinity in the printed thin films as hypothesized (Fig. 2a). We further quantified the increase in the relative degree of crystallinity (rdoc) as discussed below. The observed increase in rdoc is corroborated with the changes in molecular packing distances due to FLUENCE (Supplementary Table 1). The lamella stacking distance is shorter in the FLUENCE-printed thin film by 2–3% as compared with that of the reference film at the same printing speed, and this trend persists across the printing speeds. In addition, the lamella stacking distance decreases with an increase of printing speed in neat polymer donor films. These observations imply that the side chains become increasingly close-packed with the increase of shear rate and/or the introduction of extensional flow. The closer packing may result from either a higher degree of ordering or a higher extent of side chain interdigitation.

To quantify the observed changes in the thin film crystalline morphology, we extracted the rdoc from the diffracted peak intensity profiles of neat donor polymer thin films. This analysis follows the procedures described in previous works^55^,^56^,^57^,^58^. First of all, pole figures were obtained by plotting the (010) peak intensity as a function of the polar angle *χ*, which describes the relative orientation of the crystallites with respect to the substrate (Supplementary Figs 1 and 2). For the (010) peak, a polar angle of *χ*=0° represents ‘face-on' crystallites, whereas *χ*=90° represents ‘edge-on' crystallites. The peak intensities were normalized by the exposure time and the irradiated volume in the thin film so as to allow direct comparison among films prepared at different printing speeds. Importantly, to obtain the correct peak intensity profiles, careful background subtraction and peak deconvolution were necessary at each *χ* due to the presence of overlapping peaks (Supplementary Fig. 1). In the thus obtained (010) pole figures, the crystallite population for higher *χ* was underrepresented by the diffracted intensities. This is because only a subset of these crystallites with an in-plane orientation that satisfies the Bragg condition diffracts^57^. Therefore, geometrical corrections were needed to convert the pole figures to out-of-plane orientation distribution functions of crystallites (Fig. 2b). It is worth noting that the geometrical correction was performed based on the assumption that the crystallites are oriented isotropically in-plane, which is acceptable given that the normalized diffracted intensities are comparable along directions parallel and perpendicular to printing (Supplementary Figs 2 and 3).

Shown in Fig. 2b, the geometrically corrected intensity profiles reveal a bimodal distribution of crystallites that peaks around *χ* of 15° and 80°. Most notably, across all orientation populations and printing speeds tested, there are substantially higher fractions of crystalline domains in FLUENCE-printed thin films. In contrast, such improvement in polymer crystallinity cannot be achieved by simply varying the printing speed (Fig. 2b). In addition, increasing printing speeds re-orients the crystalline domains towards more ‘face-on' and ‘edge-on' orientations (less isotropic out-of-plane) without significantly altering the crystallinity of the thin films. We calculated the rdoc by integrating the geometrically corrected peak intensities over *χ* (from 10° to 86°) to find that FLUENCE improved the rdoc by ∼90% across the range of printing speeds tested.

### Domain size characterization via RSoXS

**Figure 3:**
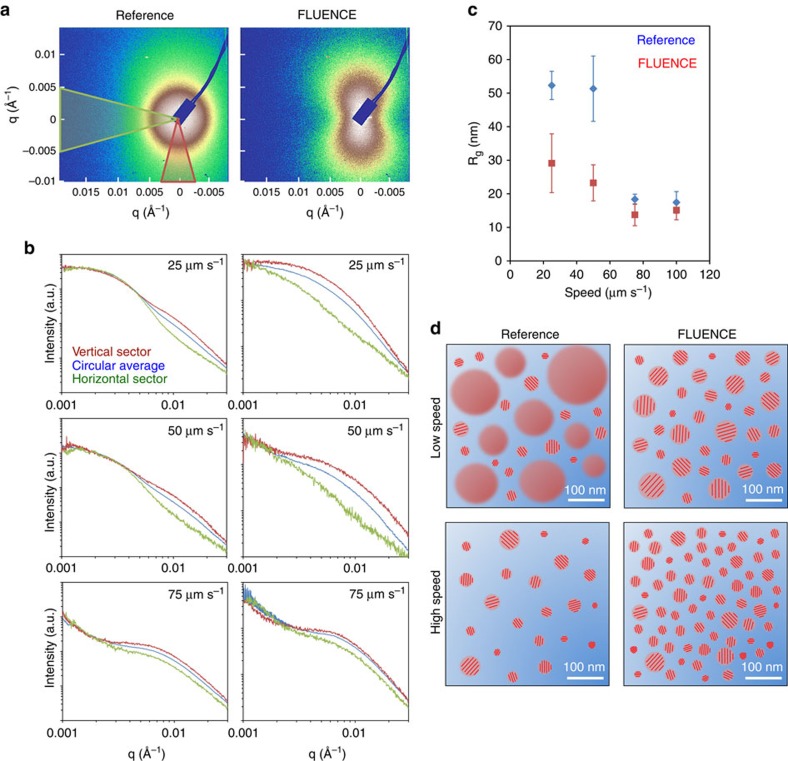
Characterization of phase-separated morphology in blend thin films via RSoXS. (**a**) Two-dimensional scattering images of reference versus FLUENCE films prepared at 25 μm s^−1^. The intensity is plotted in log scale, with white, brown, yellow, green ranging from high to low intensities. (**b**) Integrated intensity profiles of reference versus FLUENCE films prepared at various printing speeds. Data corresponding to 100 μm s^−1^ printing speeds closely resemble those at 75 μm s^−1^ and are therefore omitted. To compare the scattering anisotropy, intensity from the vertical sector (parallel to the beam polarization direction) is compared with that from the horizontal sector (perpendicular to the beam polarization direction) and the circular average. The vertical (red) and horizontal (green) sectors correspond to the highlighted regions in figure (**a**). (**c**) Radius of gyration from Guinier analysis assuming spherical domains. *R*_g_ is calculated by fitting the scattering data with I(q)=I_0_ exp(−q^2^*R*_g_^2^/3). The error bars displayed were calculated from s.e. of the fitted parameter *R*_g_. The fitted *R*_g_ values are summarized in Supplementary Table 2. The analysis was performed over a *q* range of 0.001–0.007 Å^−1^. Beyond this range, poor linearity was found in ln(I) versus *q*^2^ plot. Due to this poor linearity and the weak scattering intensity, the higher *q* feature is not quantitatively analysed but is instead illustrated schematically in **d**. The corresponding I*q*^2^ versus *q* plots (vertical sector) are shown in Supplementary Fig. 4. (**d**) Schematic illustrating the possible in-plane morphology. The schematic is simplified, and the domain connectivity is not shown. The blue medium denotes the amorphous electron-acceptor polymer, P(TP). The red domains represent electron donor PII-tT-PS5, forming amorphous (shown without red bars) and semicrystalline domains (with red bars) The semicystalline domains are not crystallites but are likely aggregates of crystallites, possibly separated by small amorphous regions.

We next characterized the domain size of the FLUENCE-printed blend films compared with the reference films to understand how our flow design impacts the phase separation. Unlike polymer-fullerene solar cells, small angle X-ray scattering based on hard X-rays is not suitable for characterizing all-polymer BHJ solar cells, due to the low contrast in electron densities between two polymer domains of similar atomic compositions and densities. Thus, we employed resonant soft X-ray scattering (RSoXS) with polarized light, where scattering contrast can be enhanced by tuning the X-ray energy through the aromatic C1s→π* resonance as opposed to the plain electron density differences ‘seen' by X-rays at harder (keV) energies^16^,^59^,^60^,^61^. The use of polarized X-rays offer sensitivity to local molecular orientation due to the anisotropic nature of molecular orbitals involved in the resonant electronic transitions. RSoXS obtained at an off-resonant energy of 270 eV did not yield scattering intensities above the background (other than speckles from surface roughness), whereas at a resonant energy of 283.5 eV, markedly different scattering profiles emerged as seen by comparing FLUENCE-printed films with the reference films prepared at the same conditions (Fig. 3, Supplementary Fig. 4). At low printing speeds, the application of FLUENCE substantially enhanced the scattering anisotropy when comparing the scattering profiles parallel and perpendicular to the beam polarization direction (Fig. 3a,b). Such anisotropy is insensitive to sample in-plane rotation, indicating that the polymer chains have local correlation in their orientation alignment (over tens of nanometres), but are globally isotropic. In the reference films, two characteristic length scales were revealed, one isotropic (low *q*) and the other anisotropic (high q). In comparison, FLUENCE-printed films exhibited one dominant length scale with a broad distribution and anisotropic scattering profile throughout the investigated *q* range. At the same time, the dominant scattering feature shifted to higher q. At high printing speeds, the isotropic scattering features diminished in the reference films and the impact of FLUENCE became less obvious other than modestly shifting the scattering feature to higher *q* (Fig. 3).

The scattering anisotropy can result from multiple factors. Crystallinity in donor polymer domains alone (randomly dispersed in the amorphous acceptor medium) can cause such local scattering anisotropy because of the alignment of molecular orbitals and therefore the transition dipole moment within the (semi)crystalline domains. Correlation of molecular orientation in closely spaced amorphous domains can also cause such scattering anisotropy^20^. We speculate that in our case, the domain crystallinity is the primary contributor to anisotropy. This is because the observed scattering data feature broad shoulders without well-defined peaks, indicating poor spatial correlation between domains (no well-defined average domain-domain spacing). Therefore, molecular orientation correlation between domains is impossible. In addition, because the matrix polymer is expected to assume randomly shaped domain boundaries, there is no mechanism to cause inter-domain orientation correlation in our system. On the other hand, the crystallinity of donor polymer domains is shown in the GIXD data (Fig. 2). On the basis of this rationale, we infer from the RSoXS observations that FLUENCE ‘converted' large amorphous donor polymer domains (corresponding to the isotropic scattering feature at low *q* in the reference films) to smaller (semi)crystalline domains (corresponding to the anisotropic scattering feature at higher *q* in the FLUENCE films) at low speeds. This inference is consistent with the GIXD results and our hypothesis of flow-induced nucleation. At higher speeds, the amorphous content was reduced for the reference films, probably due to faster solvent evaporation per unit volume of ink solution and therefore higher nucleation density. In this case, the scattering anisotropy is apparent in the reference films and was therefore insensitive to FLUENCE.

To quantify the changes in the characteristic length scale, we applied Guinier analysis to extract the apparent ‘radius of gyration' *R*_g_ (ref. 62), which in our case likely convolutes both the domain size and, to a lesser degree, the domain spacing. The model I(*q*)=I_0_ exp(−*q*^2^*R*_g_^2^/3) for dispersed spherical domains describes the scattering data well within the *q* range of 0.001–0.007 Å^−1^ for low speeds and 0.004–0.011 Å^−1^ for high speeds, covering the main scattering features in both cases. The analysis shows that FLUENCE effectively reduced *R*_g_—the length scale describing large scattering features in the blend films (Fig. 3c). At low printing speeds, FLUENCE reduced *R*_g_ by as much as 50%. However, at higher speeds, the reduction was only 15–25%. In the reference films printed at low speeds, there is a second length scale of ∼15–20 nm, likely arising from small, (semi)crystalline domains. An alternative analysis assuming a dominant role of structure factor^16^,^60^ is given in Supplementary Fig. 4, which yields the same trend as that from the Guinier analysis. Putting these analyses together with the GIXD results, we illustrate a possible albeit highly simplified picture of the film morphology in Fig. 3d, without considering inter-domain connectivity or complex domain shapes. In summary, FLUENCE transforms large amorphous domains of the donor polymer into smaller (semi) crystalline domains at low printing speeds, thereby increasing the rdoc (according to GIXD) and enhancing the scattering anisotropy (according to RSoXS). At higher printing speeds, FLUENCE moderately reduces the domain size only (RSoXS) yet markedly enhances rdoc (GIXD), possibly by increasing the area density of small (semi) crystalline domains. In both cases, the proposed morphology models are consistent with the mechanism of flow-induced nucleation.

### Surface roughness of printed thin films

**Figure 4:**
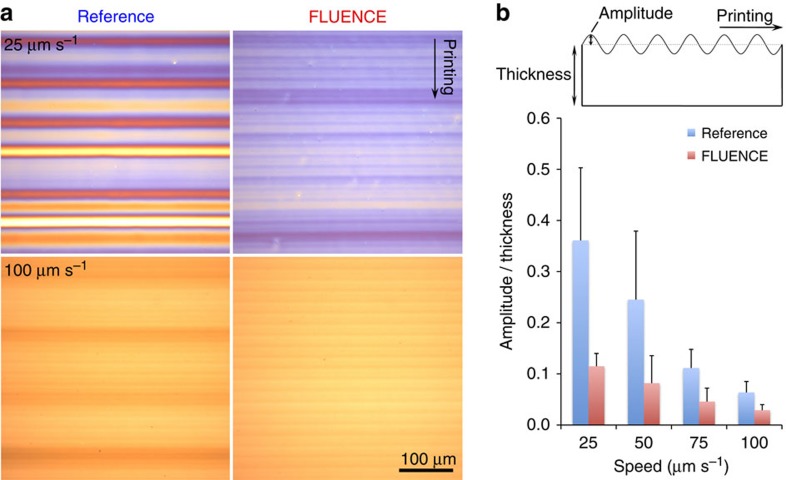
Comparison of surface roughness between FLUENCE-printed and reference thin films. (**a**) Optical micrographs of blend films printed with flat (left) and micropillar-patterned (right) blades at 25 (top) and 100 (bottom) μm s^−1^. All images share the same scale bar as shown. Wavy, periodic surface patterns are observed with pitches of 120–200 μm and 20 μm, respectively. (**b**) Reduction in surface roughness of FLUENCE-printed thin films as compared with the reference films, normalized by the film thickness. The *y* axis plots amplitude of the surface patterns over the thickness of the film, as illustrated in the schematic above the plot. The film thicknesses are 124±5, 96±8, 50±8, 30±2 nm for FLUENCE-printed films at 25, 50, 75 and 100 μm s^−1^, respectively. The error bars were calculated from five different thickness measurements across the thin films. Both the amplitude and the thickness were measured by profilometry.

In addition to enhancing crystallinity and reducing domain size, FLUENCE also lowers the film roughness significantly (Fig. 4). We observed that solution printed polymer thin films frequently exhibit wavy surface textures. These height undulations align to form stripe patterns, with their long axis perpendicular to the printing direction (Fig. 4a). Such patterns are not unusual and have been observed during the convective assembly of colloidal particles^63^,^64^ and the solution printing of crystalline ultrathin films^50^. This pattern formation was attributed to the stick-slip motion of the meniscus, wherein the ‘sticking' is caused by film deposition at the meniscus front^63^. These surface textures can cause shorting between the top and bottom electrodes and lead to large device-to-device variation. The application of FLUENCE reduced the amplitude of surface undulations by 2–3 times (Fig. 4b) and brought this to below about one tenth of the film thickness across the speed range tested. This dramatic improvement in film smoothness may be attributed to the flow-induced perturbations that reduce the meniscus pinning. We later show that such improvement is important for repeatable device performance and high device success rate.

### Solar cell device performance

**Figure 5:**
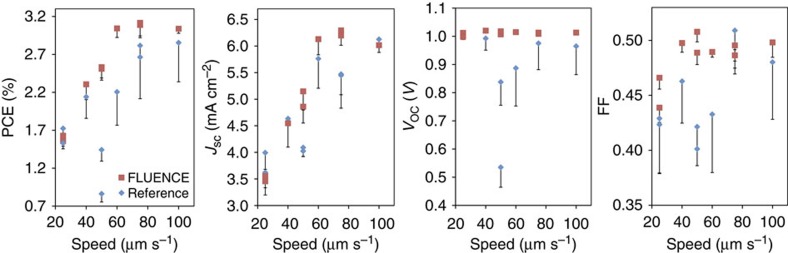
Comparison of solar cell device performance between FLUENCE and reference films. The error bars are calculated from ∼10 devices. The printing speeds range from 25–100 μm s^−1^. The solution used is a 7 mg ml^−1^ chlorobenzene solution with 1:1 donor to acceptor weight ratio. Shown in the figure are power conversion efficiency (PCE), short-circuit current (*J*_*SC*_), open circuit voltage (*V*_*OC*_) and fill factor (FF) from left to right. For the effect of the printing speed, *J*_SC_ increases with an increase in printing speed due to the optimized film thickness. At lower speed, the films are too thick (124 nm) for the efficient charge carrier collection at the electrodes. For the same reason, the FF is also low at low printing speeds. At higher printing speed, the film thickness decreases and the recombination of free charge carriers is suppressed. However, at the same time, the light absorption is reduced. Therefore, *J*_SC_ reaches the maximum at printing speed of 75 μm s^−1^ for FLUENCE-printed films. Corresponding *J–V* curves and EQE are shown in Supplementary Fig. 6. The highest performing FLUENCE-printed devices are compared with the highest performing spin-coated devices in Supplementary Table 3.

To understand the impact of the observed morphology changes on solar cell efficiency, we fabricated devices in an inverted geometry with ZnO/ITO as the electron collecting electrode and the MoO_3_/Al as the hole collecting electrode (Methods). With FLUENCE, the overall PCE significantly improved across the speed range tested. The device-to-device variation was also reduced considerably, evidenced by the much smaller s.d. (Fig. 5). The enhancement in PCE had contributions from all three factors: the short-circuit current (*J*_SC_), the open circuit voltage (*V*_OC_) and the fill factor (FF). First, the FLUENCE-printed film exhibited higher overall fill factor. The extent of enhancement reached as high as 25%. This can be attributed to the higher degree of crystallinity in FLUENCE-printed thin films, which reduces the non-geminate charge carrier recombination by enhancing charge transport^10^,^17^,^18^,^19^,^22^. In addition, the improved film smoothness may also help lower recombination due to better contact with the electrode as compared with the reference films. Second, the FLUENCE-printed thin films exhibit moderate improvement in *J*_SC_. Generally, the smaller domain size and higher crystallinity of the donor polymer phase are expected to facilitate charge generation by creating more interfacial area within the exciton diffusion length for dissociation. The enhanced crystallinity may also increase the exciton diffusion length within the donor polymer domains^23^,^24^,^25^. However, the extent of enhancement in *J*_SC_ could be complicated by other factors such as domain connectivity and interfacial orientations, which may mitigate any increase of *J*_SC_ resulting from the significant reduction in domain sizes at low printing speeds. Third, the *V*_OC_ of FLUENCE-printed films maintained a highly reproducible output at ∼1 volt, whereas that of the reference films varied substantially across various printing speeds. We attribute this improvement to the much smoother film texture in FLUENCE-printed devices, which helps to reduce leakage current between the electrodes at thinner regions. All three factors combined led to a pronounced improvement in PCE. The highest PCE reaches 3.2%, which is the best performance reported so far for solution printed all-polymer solar cell devices.

Furthermore, we demonstrate that FLUENCE is applicable to higher printing speeds as well. Shown in Supplementary Table 4, when printed at 500 μm s^−1^ from a chloroform solution, the FLUENCE-printed films exhibited higher short-circuit current and fill factor. As a result, the PCE increased by 43% on average. Correspondingly, the degree of crystallinity of the FLUENCE-printed films is substantially higher than that of the reference film, by ∼50% (Supplementary Fig. 7). The FLUENCE-printed films also exhibit much lower film roughness (Supplementary Fig. 8) at the speeds tested.

## Discussion

On the basis of the morphology characterization results, we propose the following picture of structural evolution and discuss the impact of FLUENCE on the phase separation mechanism. At low speeds, the meniscus drags out a thick liquid film^65^, and the drying rate per solvent volume is low. As the solvent drying progresses, the polymer concentration in the liquid film slowly reaches the miscibility limit, inducing liquid-liquid phase separation between donor-rich and acceptor-rich domains in the solution. The mechanism of liquid-liquid phase separation is inferred from the presence of large amorphous domains as a dominant feature in the reference films. As the solvent evaporation continues, a fraction of the donor-rich domains is able to overcome the nucleation barrier and crystallize before the rest of the film is ‘frozen' into amorphous domains when the solvent completely evaporates (only the donor polymer crystallization is discussed here since acceptor polymer is found to be amorphous via GIXD). This crystallization step following the liquid-liquid phase separation gives rise to small, semicrystalline domains coexisting with large, amorphous domains observed in the reference films prepared at low printing speeds. Application of FLUENCE pre-stretches and aligns the polymer chains through extensional flow and shear flow, respectively, thereby lowering the entropic barrier to donor polymer nucleation. By lowering the crystal nucleation barrier, FLUENCE may have altered the mechanism of phase transition from liquid-liquid phase separation to crystallization-induced phase separation^10^. Such change in the phase transition mechanism is plausible because of the absence of large amorphous domains in the FLUENCE-printed films (since there are no isotropic, low *q* scattering features in RSoXS). Due to this flow-induced nucleation, the large amorphous domains in reference films are replaced by small semicrystalline domains in the FLUENCE-printed films (RSoXS), resulting in higher degree of crystallinity overall (GIXD).

At higher printing speeds, the liquid film thickness decreases (it is inversely correlated to the printing speed)^65^, leading to a faster drying rate per volume of solution and therefore larger driving force for crystal nucleation. As the ink concentration rapidly surpasses the solubility limit, nucleation of donor polymer domains occurs. The nucleated donor polymer domains grow to push out the acceptor polymer into amorphous domains, thereby inducing microphase separation. By this mechanism of crystallization-induced phase separation, the resulting domain size in the reference films is smaller as compared with the case of liquid-liquid phase separation. The shift in phase transition mechanism may explain the disappearance of the isotropic scattering at lower *q* (Fig. 3b) and the sudden drop in *R*_g_ from lower (<50 μm s^−1^) to higher printing speeds (>50 μm s^−1^) (Fig. 3c). In this regime of printing speed, application of FLUENCE does not alter the phase transition mechanism and instead, simply increases the nucleation density via flow-induced nucleation. By this mechanism, the domain size is mainly determined by the crystal growth rate and the drying time and the impact of increased nucleation density on domain size is much reduced. Nonetheless, FLUENCE still enhances the degree of crystallinity substantially (∼90% at all speeds tested) by increasing the density of crystalline domains. In both cases, the morphological changes from FLUENCE led to the improve device performances as discussed above.

According to our hypothesis of flow-induced nucleation (discussed at the beginning), the effect of FLUENCE on morphology evolution is critically dependent on the extent of enhancement in extensional strain rate and the shear rate, which act to stretch and align the polymer chains. The flow-induced conformation change and increased orientation ordering are expected to lower the free energy barrier to nucleation^44^, thereby increasing the nucleation density and leading to the morphology changes we observed. To test this hypothesis, we increased the pillar gap (along the *x* axis) from 1.2 to 15 μm, and the row spacing (along the *y* axis) from 2.3 to 50 μm, thereby reducing the maximum extensional strain rate and shear rate by ∼170 and 60 times, respectively, (estimated from fluid simulations). As a result, the previously observed improvements in solar cell device performances were diminished (Supplementary Fig. 5), which supports our hypothesis.

The proof-of-concept study we have described opens up new avenues for controlling the polymer solar cell morphology during solution printing. Given the sensitivity of molecular conformation to flow field and the importance of mass transport during phase transition, we expect that our concept can extend beyond all-polymer solar cell systems and be applicable to a wide range of solution printed functional materials where morphology control is crucial to device performance.

## Methods

### Materials

All the polymers were synthesized according to previously reported procedures^15^. The donor polymer (PiI-tT-PS5) was purified via preparation size exclusion chromatography at room temperature with chloroform as the solvent in concentration of 7 mg ml^−1^. The molecular weight and polydispersity index of all polymers were measured by high temperature gel permeation chromatography at 160 °C with 1,2,4-trichlorobenzene as the solvent and polystyrenes as the calibration standards.

### Fluid simulation and blade fabrication

Fluid simulations were performed using the COMSOL (version 4.4) Multiphysics with the computational fluid dynamics module. For the simulation geometry, hexagonal pillar arrays normal to the surface were generated with a lattice constant of 2.67 μm. The side of the hexagon is 1 μm in length, and the pillar height is 4.5 μm, all measured from scanning electron microscopy images. The corners of the micropillars are rounded with a radius of curvature (fillet radius in COMSOL) of 0.1 μm. The tilt of the blade during printing is set as 8°. The gap of the first row of pillars to the bottom substrate is set to 0.5 μm. For the flat blade, the pillars were simply removed, leaving in place all other spatial dimensions. To simulate the shearing motion, the bottom substrate was set as a sliding wall with speed of −50 μm s^−1^. The two side walls were set as periodic boundary conditions. The inlet mass flow rate was set to equal the solvent evaporation rate. From the measured film thickness we calculated the solvent evaporation rate by applying a mass balance^65^. The normal mass flow rate at the inlet is calculated as 4.218 × 10^−12^ kg s^−1^ at the shearing conditions specified in the solar cell device section (corresponding to printing speed of 50 μm s^−1^, with a width of the simulation box of 5.34 μm). The outlet mass flow rate was set as the inverse of inlet to satisfy the mass balance. Simulations were performed using the laminar flow module to solve the steady-state Navier–Stoke equation. Given the diluteness of the polymer solutions, we estimated that shear thinning is minimal at such low concentration and viscosity (∼13 mPa s^−1^) based on a power law dependence of viscosity on strain rate. Therefore, the fluid is assumed to be Newtonian in COMSOL simulations. The initial conditions were set to zero velocity and zero pressure in the fluid, with the no-slip condition at all walls aside from the bottom substrate. The element size for the physics-controlled mesh was set to ‘normal' for the hexagonal blade simulation and ‘fine' for the flat blade. The MUMPS direct solver produced solutions in 9 h and 6 min for the former, and the PARADISO direct solver took 40 min for the latter. The simulations were run on the Stanford University's Sherlock computing cluster.

For blade fabrication, a silicon mould was prepared first by patterning a photoresist mask with a Cr mask on top of silicon wafer using standard photolithography followed by CF_4_ reactive ion dry etching. The silicon was etched by 5–10 μm. The patterned blade was then rinsed with acetone to remove the photoresist layer. The blade was then plasma-activated for 1.5 min at 150 W and 150 mTorr O_2_ and immersed in a 0.1 vol.% OTS/trichloroethylene (anhydrous) solution for 20 min at room temperature. After rinsing with toluene and isopropanol, the wafer was annealed at 120 °C for 20 min.

### Morphology characterizations

GIXD images were collected in reflection mode with a two-dimensional area detector and the sample in a helium atmosphere at beamline 11-3 of the Stanford Synchrotron Radiation Lightsource. The sample to detector distance was 400 mm, and the incidence angle was 0.12°. The X-ray wavelength was 0.9758 Å, corresponding to a beam energy of 12.7 keV. Two sets of samples were prepared, one of neat donor polymer films and the other of blend polymer films (1:1), both solution sheared from 7 mg ml^−1^ chlorobenzene solutions at 50 °C at various printing speeds. The substrate used for both sets of samples was ZnO-coated bare Si wafer with a layer of native oxide. The method for preparing the ZnO layer is described below in the device testing section. Samples were cut into 5-mm wide strips for GIXD measurements. The method for extracting the relative degree of crystallinity is described in Supplementary Figs 1 and 2, their captions in the supporting information and Fig. 1 in the main text.

RSoXS data were collected at the Advanced Light Source beamline 11.0.1.2 in transmission geometry^66^. For sample preparation, native oxide Si wafers were first treated in ultraviolet-ozone for 20 min followed by spin-coating of poly(sodium 4-styrenesulfonate) from its 10 wt% aqueous solution at 5,000 r.p.m. for 30 s. The substrates were then baked in air at 80 °C for 10 min to remove residual water. The PII-tT-PS5:P(TP) (1:1) blend films were solution printed on the poly(sodium 4-styrenesulfonate)-coated Si wafer from 7 mg ml chlorobenzene solution at 50 °C, floated off in deionized water, and then picked up with a 1 × 1 mm, 100-nm-thick Si_3_N_4_ membrane supported on a 5 × 5 mm, 200-μm-thick Si frame (Norcada Inc.). The film was then dried in air before being transferred into the vacuum chamber for RSoXS. We swept the beam energy from 270 to 290 eV, with a 10 s exposure time per scan. The highest scattering contrast was found to be at 283.5 eV. Scattering patterns were collected on a two-dimensional charge-coupled device camera in vacuum and cooled to −44 °C (Princeton Instrument PI-MTE). Data analysis was performed using the Nika package supported in the Igor Pro environment (http://usaxs.xray.aps.anl.gov/staff/ilavsky/nika.html).

Profilometry was performed to measure the surface texture and the film thickness using the Bruker Dektak 150 profilometer. The stylus force was set to 1 mg. Scan range was set to 1,000 μm. For film thickness measurements, the film was scratched using the tip of the tweezers so that only the film was scratched and not the substrate beneath. The scan was run across the scratch. This measurement was repeated 5–10 times to obtain the average and s.d.

### Solar cell fabrication and testing

Glass substrates coated with patterned ITO with a sheet resistance of 13 Ω/□ were purchased from Xin Yan Technology Lt. Before device fabrication, the ITO/glass substrate was ultrasonicated sequentially in acetone, detergent, deionized water and isopropanol. The substrate was dried in a vacuum oven at 80 °C for 10 min and then cleaned by a 20-min ultraviolet-ozone treatment. A solution of zinc hydroxide in ammonium was spin-coated onto the ITO surface at a speed of 5,000 r.p.m. for 30 s. The film was baked at 90 °C for 10 min in air to form a 10-nm-thick ZnO film. The polymers were dissolved in chlorobenzene and stirred for 3 h. The concentration was 7 mg ml^−1^ for PiI-tT-PS5 and P(TP) combined (1:1 ratio by weight). The solution was filtered with a 0.45 μm polytetrafluoroethylene syringe filter before shearing. Solution shearing was performed at a substrate temperature of 50 °C, with a gap size set as the same as the height of the micropillars on the printing blade. A side camera was used in transmission geometry to align the blade to the substrate. The blade was tilted by 8°, and the printing speeds ranged from 25–100 μm s^−1^. Reference films were made at the same conditions using the flat blade at a gap of 30 μm. After film preparation, the samples were transferred to a vacuum evaporator for electrode deposition. A MoO_3_ layer (15 nm) followed by a Ag layer (150 nm) were thermally deposited at a pressure of 8 × 10^−6^ Torr. The device active area is 4.0 mm^2^. All the devices were tested inside a nitrogen glove box after encapsulation under AM 1.5G illumination with an intensity of 100 mW cm^−2^ (Newport Solar Simulator 94021A) calibrated by a Newport-certified silicon photodiode covered with a KG5 filter. The photodiode active area was 6.63 mm^2^, which is comparable to our device area of 4.0 mm^2^. The *J–V* curves were recorded with a Keithley 2400 semiconductor analyzer.

## Additional information

**How to cite this article:** Diao, Y. *et al.* Flow-enhanced solution printing of all-polymer solar cells. *Nat. Commun.* 6:7955 doi: 10.1038/ncomms8955 (2015).

## Supplementary Material

Supplementary Information Supplementary Figures 1-8, Supplementary Tables 1-4 and Supplementary References
