# Supplementary material for: Flow-enhanced solution printing of all-polymer solar cells
Source: Nat Commun. 2015 Aug 12;6:7955. doi: 10.1038/ncomms8955 (PMC4557117; doi:10.1038/ncomms8955)
Supplement: Supplementary Information — Supplementary Figures 1-8, Supplementary Tables 1-4 and Supplementary References [file ncomms8955-s1.pdf]

Supplementary Figures

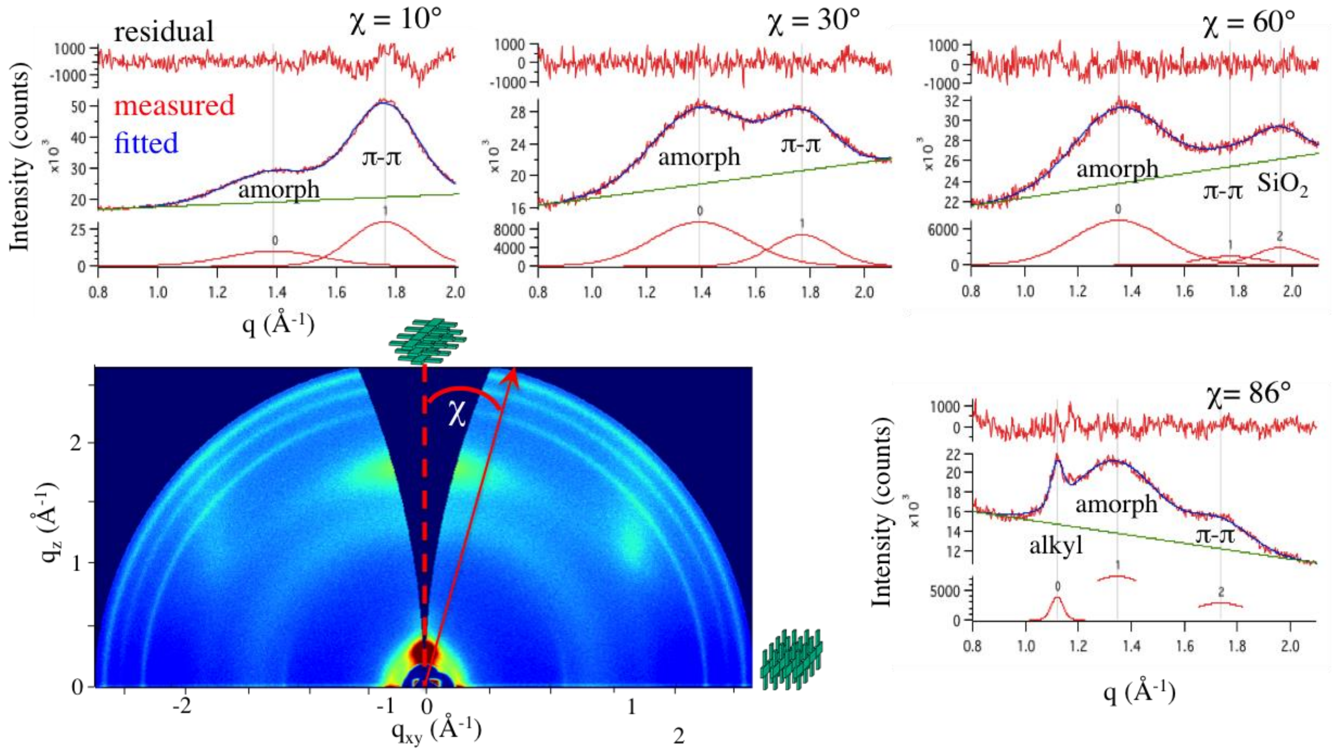

Supplementary Figure 1. Illustrations of peak deconvolutions for extracting the (010) π-π stacking peak intensities as a function of the pole angle χ. The thus constructed pole figures are shown in Supplementary Figure2. Cake segments with an angular breadth of 2° were taken centered at χ = 10, 15, 20, 25, 30, 35, 40, 50, 60, 70, 80 and 86°. Peak deconvolutions were performed at a q range between 0.8 and 2.0 $Å^{-1}$. Four examples of peak deconvolutions are shown, with a linear background represented as green lines. The measured and fitted peak intensity profiles are colored red and blue respectively. At χ = 50°, 60°, the weak π-π stacking peak partially overlays with the scattering from the SiO_2/Si substrate, resulting in larger standard deviations of the fitted peak area and consequently, larger error bars in Figure 2.

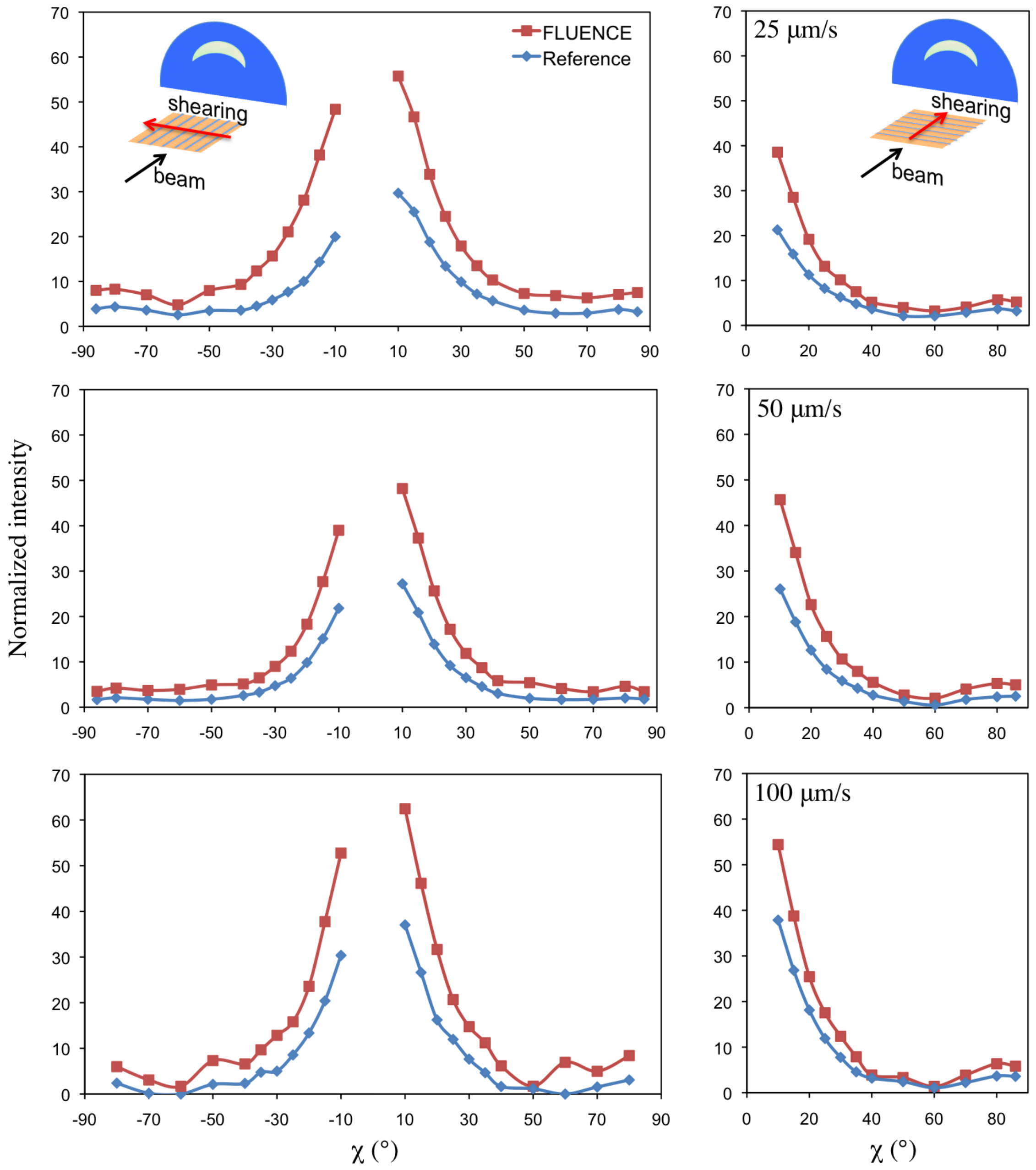

Supplementary Figure 2. Pole figures for the (010) reflection from neat PII-tT-PS5 films at printing speeds from 25, 50 to 100μm/s. Left and right columns correspond to the results in "perpendicular" and "parallel" directions respectively, defined in terms of the shearing direction with respect to the incident beam direction as illustrated in the insets. The intensity of the (010) peak as a function of the pole angle χ was obtained by performing peak deconvolutions illustrated in Supplementary Figure1. The normalized intensity was obtained by dividing the detector counts by the exposure time, average film thickness, and the beam path length on the sample. The normalized intensities are comparable for the perpendicular and the parallel configurations at the same printing speed. Therefore, the printed films are approximated as isotropic in-plane and oriented out-of-plane, or as "2-D powder" textures, for performing the geometrical correction, $\sin(\chi)I(\chi)$, which was done in order to obtain the orientation distribution functions shown in Figure 2b. The "parallel" configuration yields symmetric diffraction patterns and therefore only half of the pole figure is shown. The origin of the asymmetry in the "perpendicular" configuration is explained in Supplementary Figure3.

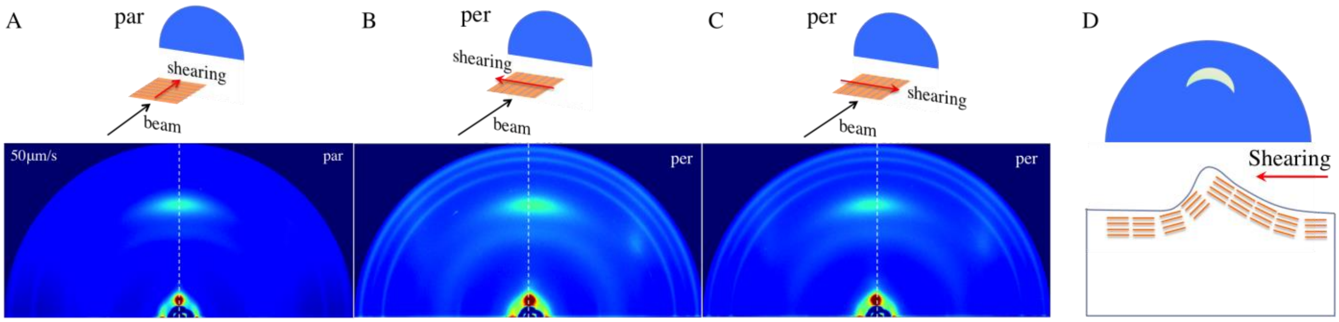

Supplementary Figure 3. Asymmetry of peak intensities observed in the “perpendicular” configuration (with shearing direction perpendicular to the incident beam). Diffraction patterns in the parallel and two opposite perpendicular directions are shown in (A-C). Tilt is observed for both the π-π stacking (010) and the lamella stacking (100) peaks, and the tilting direction depends on how the film is oriented with respect to the beam. The origin of this asymmetry (tilt) is attributed to the surface texture of the film, as illustrated in (D). The orange-colored lines represent the π-π stacking planes of a crystallite. This depiction of the surface texture is consistent with the results from profilometry. Diffraction patterns from films printed at 50µm/s are shown and are representative of all printing speeds in terms of this asymmetry.

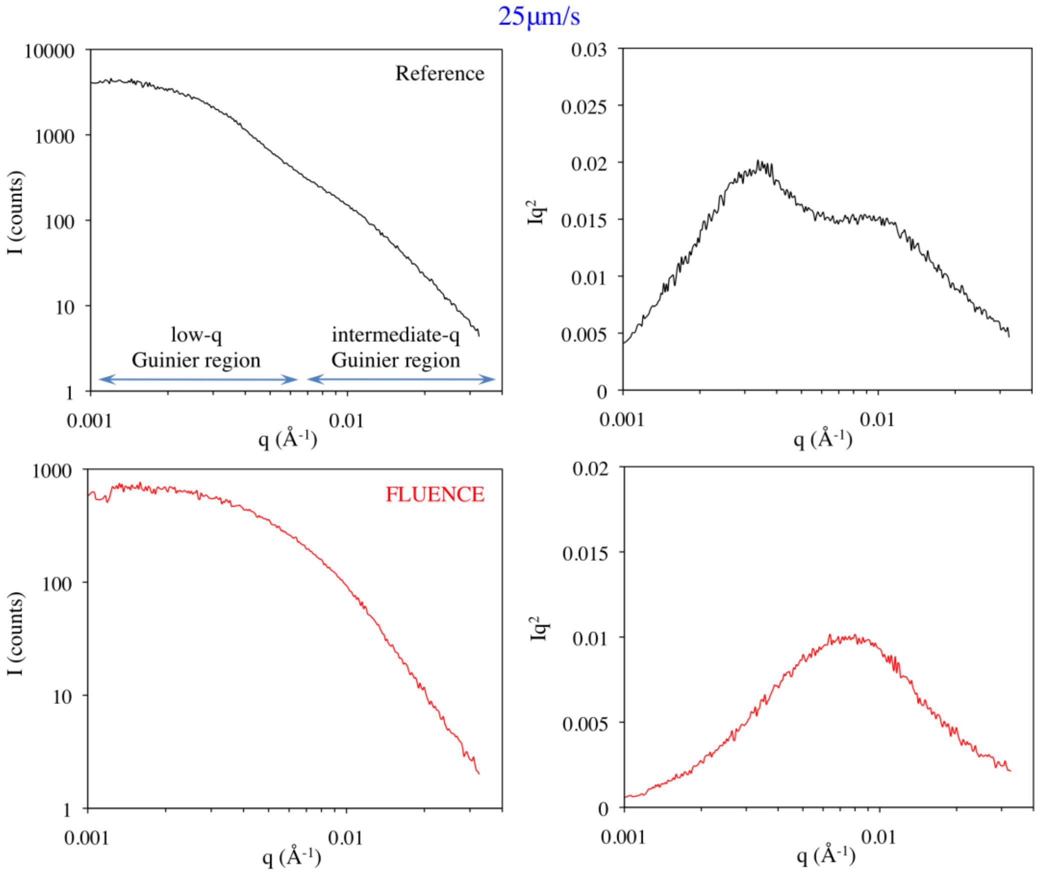

50µm/s

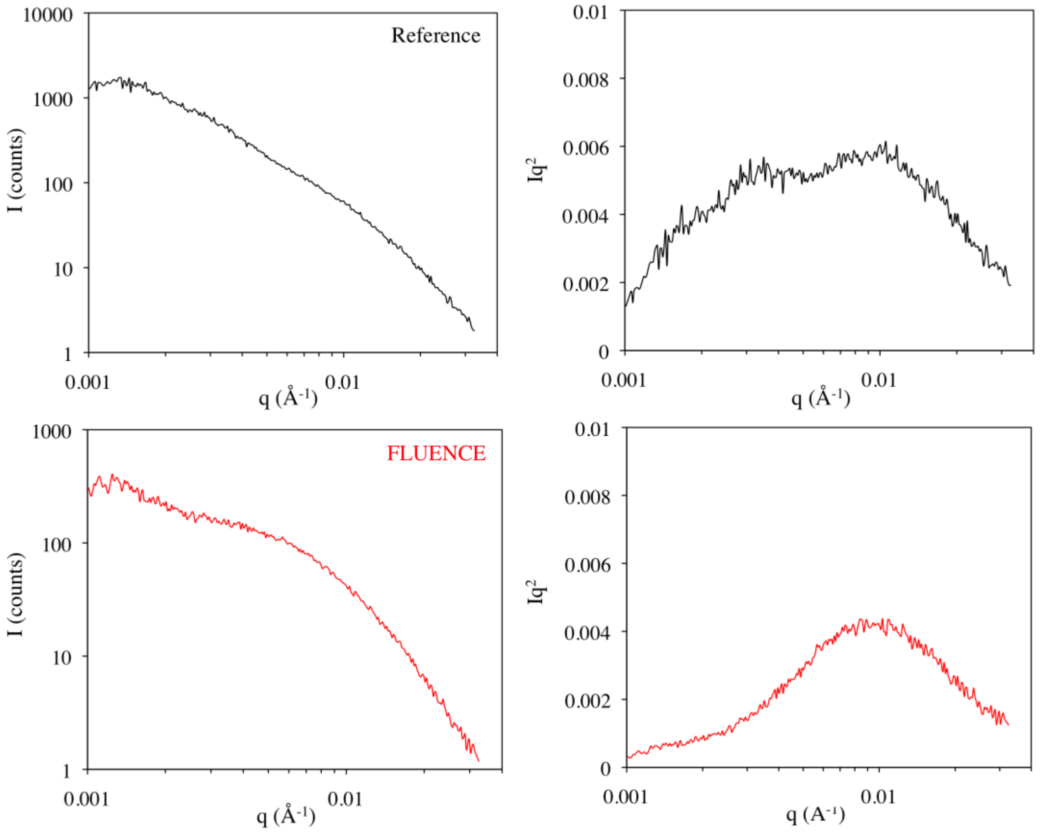
Reference
FLUENCE
I (counts)
Iq²
q (Å⁻¹)
10000
1000
100
10
1
0.001
0.01
0.008
0.006
0.004
0.002
0

75µm/s

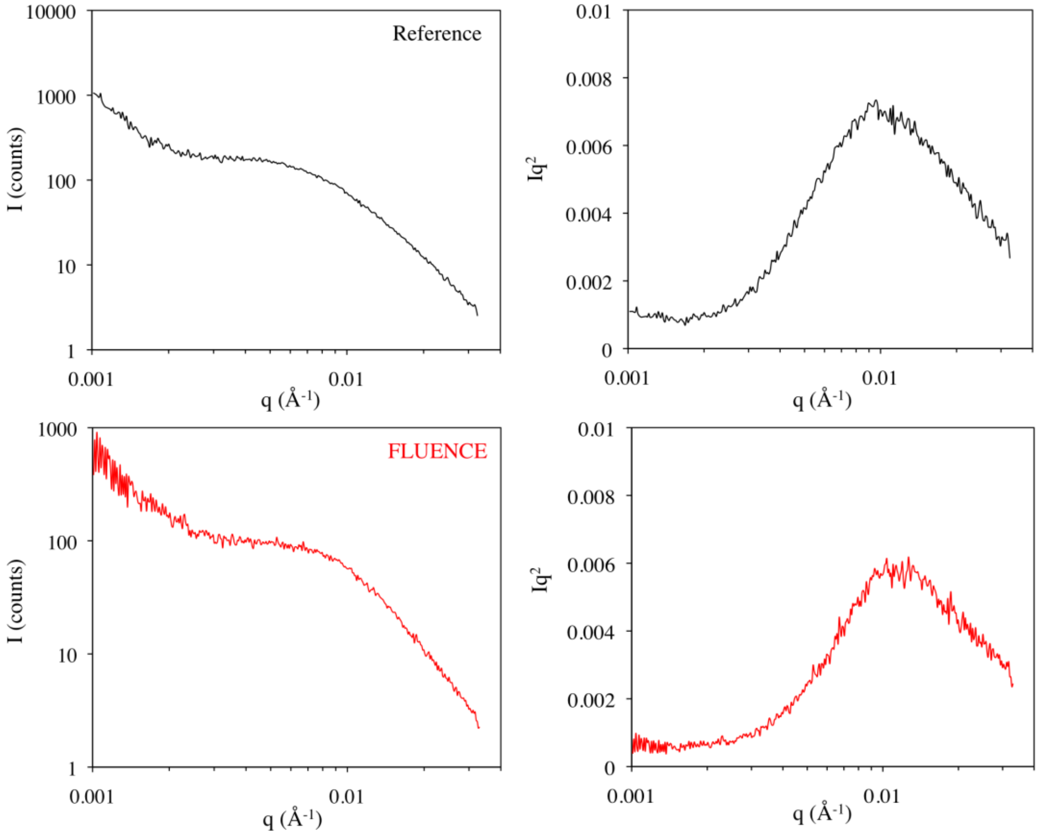
Reference
FLUENCE
I (counts)
Iq²
q (Å⁻¹)
10000
1000
100
10
1
0.001
0.01
0.008
0.006
0.004
0.002
0

Supplementary Figure 4. Azimuthally angle-averaged RSoXS data of blend films printed at various speeds. Background subtraction was applied using the scattering data from a bare Si_3N_4 membrane at the same energy as the background scattering. The left column compares FLUENCE-printed (red) with reference films (black) in I vs. q plots on log-log scale and the right column in Iq^2 - q plots in semi-log scale. The Iq^2 - q plots are useful for extracting inter-domain spacing by applying d=2π/q at peak positions, when the structure factor plays a dominant role as opposed to the form factor.[1] In this work, we applied the Guinier analysis instead. Nonetheless, the Iq^2 - q plots are provided as a reference. The scattering data were collected at a resonant beam energy of 283.5 eV. The scattering data at 100 µm/s is comparable to that printed at 75 µm/s.

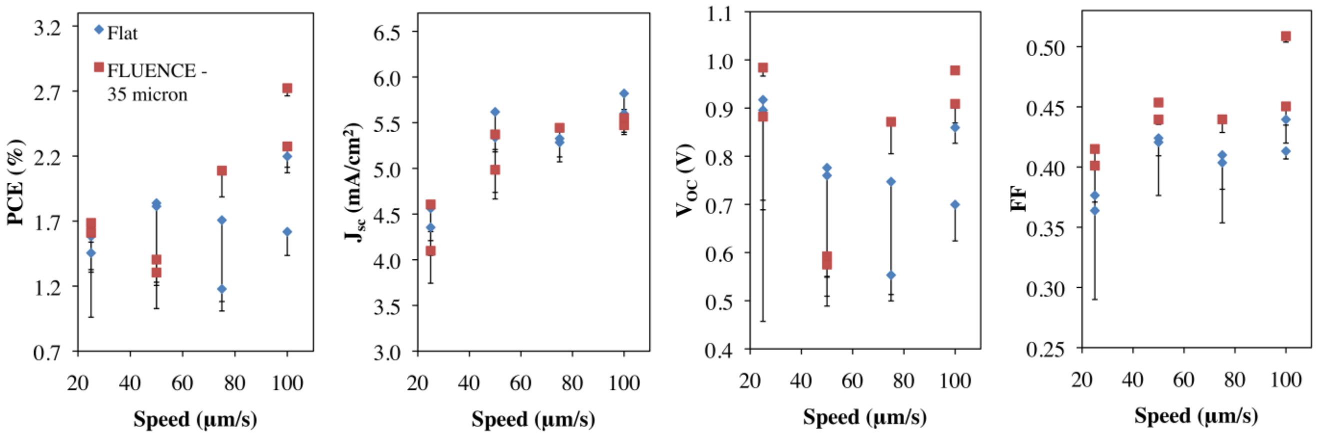

Supplementary Figure 5. Comparison of solar cell device performance between FLUENCE-printed films and reference films across various printing speeds (25-100µm/s). The geometry of the micropillars is as follows: the 35-µm-wide, 42-µm-tall pillars form hexagonal arrays with a 15-µm gap size between the pillars and 50-µm row spacing. Shown in the figure are power conversion efficiency (PCE), short circuit current (J_{sc}), open circuit voltage (V_{oc}), and fill factor (FF) from left to right. The error bars are calculated from ~10 devices. The solution used is a 7mg/mL chlorobenzene solution with 1:1 donor to acceptor weight ratio. The gap size was set to 45 µm. The substrate temperature was set to 50°C.

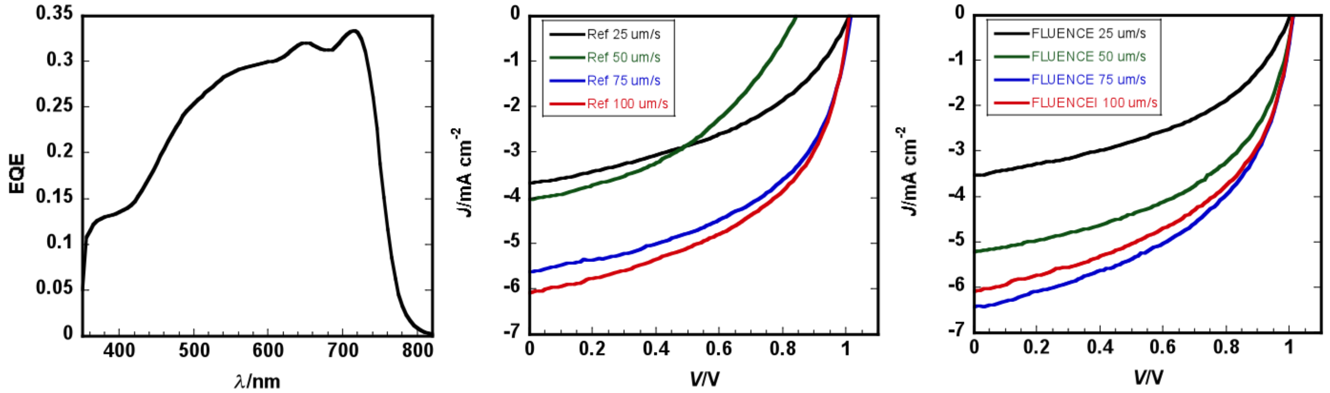

Supplementary Figure 6. Device characteristics of the printed OPV devices. The EQE (external quantum efficiency) of the highest performing device is shown on the left. The integrated current is 6.59 mA/cm^2, which matches the J_{sc} we obtained from the *J-V* curve. *J-V* curves of the devices printed at each speed are shown in the middle (reference films) and right (FLUENCE-printed films). Note that the reference films have much broader distribution in device performance (see Fig. 5). Here, only one J-V curve is shown at each condition.

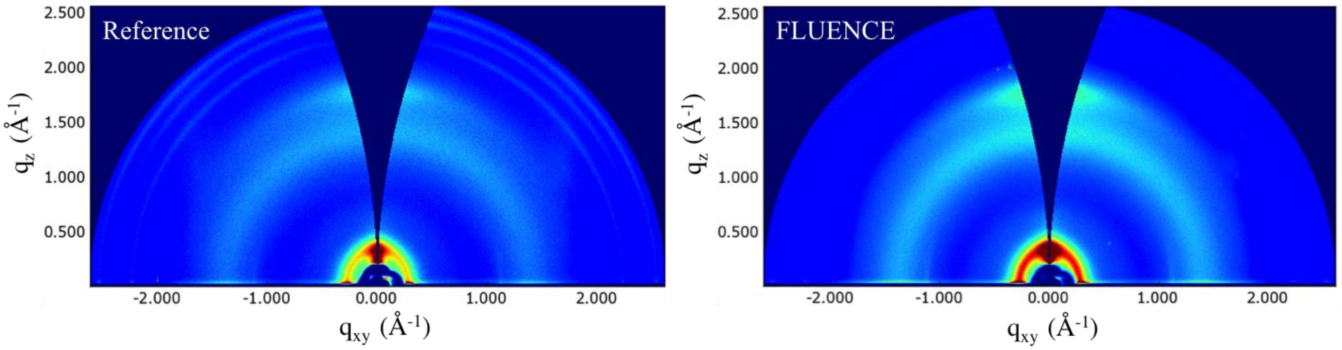

Supplementary Figure 7. Comparison of GIXD images of blend films printed at high speed (500 μm/s) from chloroform solution: reference film (left) vs. FLUENCE-printed films (right). Device performance is summarized in Supplementary Table4. The printing conditions are detailed in the caption of Supplementary Table4. The degree of crystallinity increased by approximately 50% when using FLUENCE.

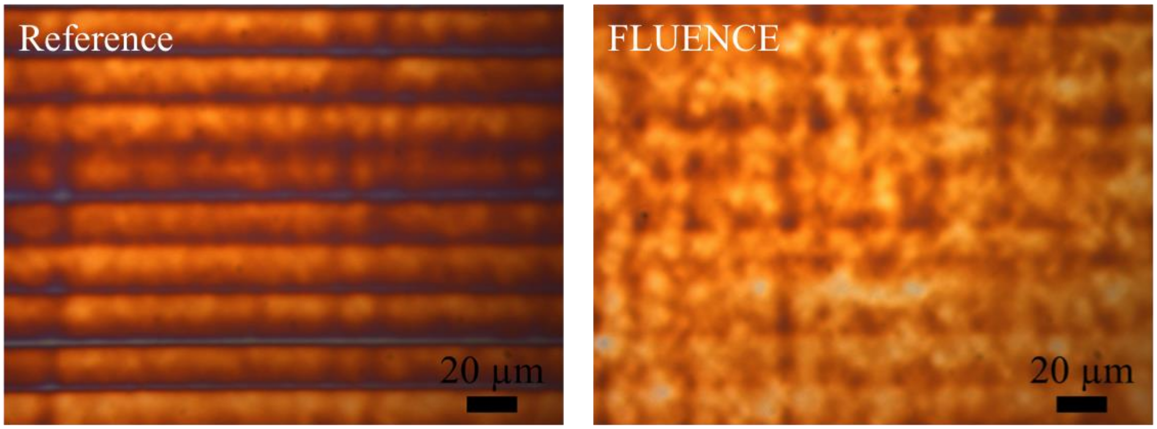

Supplementary Figure 8. Comparison of optical microscopy images between reference films and FLUENCE-printed films at high speed from chloroform. FLUENCE substantially reduced the film roughness at high printing speed.

Supplementary Tables

Supplementary Table 1. Comparison of crystal packing between FLUENCE-printed and reference films printed without FLUENCE, obtained via GIXD.

Speed (μm/s)	Printing method	Neat film				Blend film			
		q_{xy}(100) (Å^{-1})	d(100) (Å)	q_{xy}(010) (Å^{-1})	d(010) (Å)	q_{xy}(100) (Å^{-1})	d(100) (Å)	q_{xy}(010) (Å^{-1})	d(010) (Å)
25	Reference	0.309	**20.37**	1.774	3.541	0.305	20.61	1.787	3.517
	FLUENCE	0.317	**19.83**	1.775	3.540	0.307	20.47	1.785	3.519
50	Reference	0.311	**20.20**	1.766	3.559	N/A	N/A	1.786	3.519
	FLUENCE	0.318	**19.78**	1.767	3.556	N/A	N/A	1.789	3.512
100	Reference	0.315	**19.93**	1.767	3.556	N/A	N/A	1.785	3.520
	FLUENCE	0.324	**19.39**	1.767	3.555	N/A	N/A	1.787	3.517

The lamella stacking distance, or d(100), is shorter in the FLUENCE-printed thin film by 2-3% as compared to that of the reference film at the same printing speed (highlighted as bold). This trend is persistent across the range of printing speeds and is observed in both the neat and the blend films, although in the case of blend films, d(100) cannot be reliably extracted at higher speeds due to the low peak intensity and its convolution with the bright peak from the substrate reflection. In addition, d(100) is decreasing with the increase of printing speed in the case of neat films. These observations imply that the lamella stacking becomes increasingly close packed with either the increase of shear rate or introduction of extensional flow. The closer packing may have resulted from either higher degree of ordering or higher extent of side chain interdigitation. This inference is corroborated by the increased diffraction intensity of the (010) peak in FLUENCE-printed films (Fig. 2a insets). Comparing neat with blend films, the π-π stacking distances, or d(010), decreased by ~1% upon addition of the acceptor polymer P(TP). It is unclear what may have caused this. Both (010) and (100) peak positions are taken from the face-on component of the diffraction patterns. The peak positions are independent of sample orientation parallel and perpendicular to the incident beam with respect to the printing direction.

Supplementary Table 2. Characteristic length scale of microphase separation obtained from Resonant Soft X-ray Scattering (RSoXS) patterns of solution-printed polymer thin films prepared with the flat (red) and micropillar patterned (blue) blades (FLUENCE). R_g is the radius of gyration extracted from Guinier analysis.

Printing speed (μm/s)	R_{g1} (nm)	
	Flat	Hex
25	52.3 ± 4.2	29.1 ± 8.8
50	51.3 ± 9.7	23.3 ± 5.4
75	18.4 ± 1.5	13.8 ± 3.3
100	17.4 ± 3.2	15.1 ± 2.9

Supplementary Table 3. Comparison of the highest performing spin-coated and FLUENCE-printed devices.

	PCE (%)	J_{sc} (mA cm^{-2})	V_{oc} (V)	FF
PiI-tT (spun)	1.67 ± 0.08	4.10 ± 0.10	0.97 ± 0.01	0.42 ± 0.01
PiI-tT (printed)	3.42 ± 0.10	8.14 ± 0.18	0.98 ± 0.00	0.43 ± 0.01
PiI-tT-PS5 (spun)	2.75 ± 0.06	5.92 ± 0.25	0.99 ± 0.01	0.45 ± 0.01
PiI-tT-PS5 (printed)	3.27 ± 0.14	7.04 ± 0.14	1.01 ± 0.01	0.46 ± 0.02

Supplementary Table 4. Comparison of device performance at high printing speed (500 µm/s) printed from chloroform solution. Shown are the average performance followed by the highest performance in parentheses.

	PCE (%)	J_{sc} (mA cm^{-2})	V_{oc} (V)	FF
Reference	1.31(1.62)	4.28(4.86)	1.01(1.01)	0.33(0.34)
FLUENCE	1.87(2.10)	5.03(5.44)	1.01(1.01)	0.37(0.38)

Notes:

In Supplementary Table2: R_g of the small features all fall in the range of 11.8 ± 1.8 nm.

In Supplementary Table3: For FLUENCE-printed films, high performing devices are reported here (corresponding to a printing speed of 75µm/s in the case of PiI-tT-PS5 and 150µm/s in the case of PiI-tT, while keeping other conditions the same as those detailed in the Method section).

In Supplementary Table4: Solution printing was carried out using a chloroform solution with total concentration of 10 mg/mL and donor to acceptor ratio of 1:1 by weight. The printing stage was held at 50°C. The printing speed was 500 µm/s. Device performance shown is averaged over at least five different devices. It is worth noting that to print under the evaporative regime such that phase separation occurs under the influence of fluid flow, the printing speed needs to match with the evaporation rate.[2] Therefore, for high boiling point solvents, the accessible range of printing speed is small due to slow solvent evaporation. The bulk part of the work is performed using chlorobenzene. To demonstrate application of FLUENCE for higher throughput printing applications, chloroform was used here instead.

Supplementary References

1. Collins, B. A.; Li, Z.; Tumbleston, J. R.; Gann, E.; McNeill, C. R.; Ade, H., Absolute Measurement of Domain Composition and Nanoscale Size Distribution Explains Performance in PTB7:PC71BM Solar Cells. *Adv Energy Mater.* **2013,** *3* (1), 65-74.
2. Le Berre, M.; Chen, Y.; Baigl, D., From Convective Assembly to Landau-Levich Deposition of Multilayered Phospholipid Films of Controlled Thickness. *Langmuir* **2009,** *25* (5), 2554-2557.
